# Impaired memory consolidation in children with obstructive sleep disordered breathing

**DOI:** 10.1371/journal.pone.0186915

**Published:** 2017-11-02

**Authors:** Kiran Maski, Erin Steinhart, Hannah Holbrook, Eliot S. Katz, Kush Kapur, Robert Stickgold

**Affiliations:** 1 Department of Neurology, Boston Children’s Hospital, Boston, Massachusetts, United States of America; 2 Department of Psychology, University of Vermont, Burlington, Vermont, United States of America; 3 Division of Respiratory Diseases, Boston Children’s Hospital, Boston, Massachusetts, United States of America; 4 Department of Psychiatry, Beth Israel Deaconess Medical Center, Boston, Massachusetts, United States of America; 5 Department of Psychiatry, Harvard Medical School, Boston, Massachusetts, United States of America; University of Rome Tor Vergata, ITALY

## Abstract

Memory consolidation is stabilized and even enhanced by sleep (and particularly by 12–15 Hz sleep spindles in NREM stage 2 sleep) in healthy children but it is unclear what happens to these processes when sleep is disturbed by obstructive sleep disordered breathing. This cross-sectional study investigates differences in declarative memory consolidation among children with primary snoring (PS) and obstructive sleep apnea (OSA) compared to controls. We further investigate whether memory consolidation group differences are associated with NREM stage 2 (N2) sigma (12–15 Hz) or NREM slow oscillation (0.5–1 Hz) spectral power bands. In this study, we trained and tested participants on a spatial declarative memory task with cued recall. Retest occurred after a period of daytime wake (Wake) or a night of sleep (Sleep) with in-lab polysomnography. 36 participants ages 5–9 years completed the protocol: 14 with OSA as defined by respiratory disturbance index (RDI) > 1/hour, 12 with primary snoring (PS) and 10 controls. OSA participants had poorer overall memory consolidation than controls across Wake and Sleep conditions [OSA: mean = -18.7% (5.8), controls: mean = 1.9% (7.2), t = -2.20, P = 0.04]. In contrast, PS participants and controls had comparable memory consolidation across conditions (t = 0.41; P = 0.38). We did not detect a main effect for condition (Sleep, Wake) or group x condition interaction on memory consolidation. OSA participants had lower N2 sigma power than PS (P = 0.03) and controls (P = 0.004) and N2 sigma power inversely correlated with percentage of time snoring on the study night (r = -0.33, P<0.05). Across all participants, N2 sigma power modestly correlated with memory consolidation in both Sleep (r = 0.37, P = 0.03) and Wake conditions (r = 0.44, P = 0.009). Further observed variable path analysis showed that N2 sigma power mediated the relationship between group and mean memory consolidation across Sleep and Wake states [*B*_indirect_ = 6.76(3.5), z = 2.03, P = 0.04]. NREM slow oscillation power did not correlate with memory consolidation. All results retained significance after controlling for age and BMI. In sum, participants with mild OSA had impaired memory consolidation and results were mediated by N2 sigma power. These results suggest that N2 sigma power could serve as biomarker of risk for cognitive dysfunction in children with sleep disordered breathing.

## Introduction

Obstructive sleep disordered breathing (SDB) refers to partial or complete upper airway collapse that can exist along a continuum including snoring, upper airway resistance syndrome, and obstructive sleep apnea (OSA). It is estimated that 10% of children snore and 1–5% of the pediatric population has OSA[1]. The current International Classification of Sleep Disorders (ICSD) [2] specifies that an obstructive Apnea Hypopnea Index (AHI) ≥ 1 event per hour on an overnight polysomnogram (PSG) is diagnostic of OSA in the appropriate clinical context. The Respiratory Disturbance Index (RDI) is a more sensitive index of sleep disordered breathing that includes respiratory effort related arousals (any reduction in nasal airflow resulting in a 3-second arousal from sleep) in addition to obstructive apneas and hypopneas. While the ICSD specifies that OSA can be defined in adults using RDI [2], similar specifications are not offered for pediatric OSA. This is problematic, as SDB with AHI < 1 can still impact children’s academic performance and behavior [3–5]. In order to define clinically relevant disease, more data are needed to link specified levels of pediatric SDB to adverse outcomes.

Though parents and teachers frequently describe memory problems among children with SDB, neuropsychological tests have not consistently identified objective memory deficits in this population [6–8]. Such discrepancies may be because neuropsychological batteries generally test short-term memory whereas academic achievement relies on additional long-term memory function. Memory functioning can be thought of in three stages: encoding or learning of task, memory consolidation and memory recall. During the memory consolidation phase, memory traces become more stable and resistant to interference, allowing for better subsequent recall. Importantly, memory consolidation processes are sleep-dependent in adults and children [9–12] and have been shown to be impaired in adults with OSA [13]. One study to date showed that sleep-dependent memory consolidation was impaired in children with OSA[14]; however, children in this study were not evaluated for pre-existing neurodevelopmental disorders such as attention deficit hyperactivity disorder (ADHD) or learning problems that could influence results. Thus, it is unclear if OSA and/or milder SDB can directly affect memory consolidation processes in children.

Sleep-dependent memory consolidation in healthy children is associated with specific sleep architecture features including electroencephalographic (EEG) NREM stage 2 (N2) sleep spindles (12–15 Hz EEG oscillations) [12] and NREM slow oscillations (0.5–1 Hz) [15]. Whether the presence of obstructive SDB disrupts this important sleep neurophysiology in children is unknown. The current study compares declarative memory consolidation in children who have a spectrum of SDB to controls, examines if there are any group differences in sleep macro- and micro-architecture, and explores associations between these sleep measures and sleep dependent memory consolidation. We compare findings between three groups of children: controls, primary snorers (PS) and those with OSA (defined in this study as RDI >1/hour). We hypothesized that compared to controls, children with OSA would demonstrate decreased sleep-dependent memory consolidation and results would be predicted by NREM slow oscillation power and N2 sleep spindle density as represented by N2 sigma power. Given recent data showing cognitive deficits in children with even milder SDB [16–18], we also explored these hypotheses for children with PS compared to controls.

## Materials and methods

### Participants

Patients ages 5–9 years of age referred for a clinical overnight polysomnogram at Boston Children’s Hospital (BCH) Pediatric Sleep Laboratory for the evaluation of SDB were contacted to participate in this study. Twelve controls who reported good health, no sedating or alerting medication use, and whose guardians reported no snoring on the Pediatric Sleep Questionnaire (PSQ) [19], were recruited from community advertisement and BCH research website postings. Recruitment for this study occurred between 7/2013-6/2015.

Exclusion criteria for all participants included (1) significant hearing or vision loss; (2) metabolic, neurological, neurogenetic, psychiatric, learning or developmental disorders including diagnoses of Attention Deficit Hyperactivity Disorders (ADHD) and/or Autism Spectrum Disorder; (3) a previously diagnosed sleep disorder; (4) an unstable chronic medical condition such as asthma, diabetes, cystic fibrosis, or cardiac disease; (5) current use of a medication known to affect sleep, memory, or daytime vigilance (e.g., psychotropic medications, sedatives, hypnotics, stimulants). In addition, a potential control was excluded if he/she snored >25% of the night (as documented by sleep lab technician), had an obstructive RDI > 1/hour, and/or had clinically significant periodic limb movements during sleep (periodic limb movement index >5/hour) on the overnight polysomnogram.

Participants with OSA were recruited from referrals to Boston Children’s Hospital Sleep Laboratory for clinical concern of obstructive sleep disordered breathing. Recruitment was performed prior to the diagnostic sleep study so classification PS or OSA was only determined after completion of the protocol. Classification of PS was based on the presence of loud snoring at home reported by parent on the PSQ, snoring ≥25% of the PSG night reported by technician, and obstructive RDI <1/hour. Participants with OSA were defined as those with reported loud snoring at home based on PSQ responses and obstructive RDI ≥ 1/hour on the overnight PSG. Thirty-eight participants met both inclusion and exclusion criteria for control, PS or OSA groups. One control subject was excluded for findings of periodic limb movements of sleep and another controls was re-classified as PS because of near persistent snoring present on the study night. One 5-year old subject in the PS group was excluded from analysis as an outlier because memory consolidation performance extended more than 3 times the interquartile range from the edge of a box plot. In total, data from 12 PS participants, 14 OSA participants, and 10 controls were analyzed in this study.

Information on age, gender, body mass index (BMI), maternal educational level, ethnic background, and parental concerns for behavioral problems were obtained from questionnaires and medical records (Table 1). Parents/guardians completed the Child Behavior Checklist (CBCL) [20] and Conners' Parent Rating Scale–Revised: Short Form (CPRS–R:S) [21] for behavioral concerns. The study was approved by the Boston Children’s Hospital Institutional Review Board. All participants provided assent and their parents gave written informed consent. Participants were reimbursed for their participation with gift cards provided at the completion of each phase—Wake training and testing, and Sleep training and testing.

**Table 1:** Participant baseline clinical characteristics.

	OSA N = 14	PS N = 12	Controls N = 10	P-value
**Age (years)**	7.6 (1.2)	7.9(1.3)	7.2(1.5)	0.50
**BMI (kg/m**^**2**^**)**	19.8(4.6)	20.2(5.4)	15.8(1.8)	0.05
**Male Gender (%)**	10 (71.4%)	6 (50%)	6 (60%)	0.53
**White/non-Hispanic (%)**	10 (71.4%)	9 (72.4)	6 (60%)	0.38
**Maternal Education (%):**				
**High School graduate or less**	2(14.3%)	3(25%)	2(20%)	0.73
**College/College graduate**	9(64.3%)	5(41.7%)	4(40%)	0.73
**Post-graduate education**	3(21.4%)	4(33.3%)	4(40%)	0.67
**CBCL total T-score**	49.5(12.8)	55.2(8.8)	45.3(9.8)	0.14
**Conners ADHD T-score**	51.6(9.4)	53.8(11.9)	49.7(8.7)	0.65
**Pediatric Sleep Questionnaire**	8.9(3.3)	8.8(3.7)	2.8(2.9)	**<0.001**

Baseline clinical characteristics of participants. OSA group is defined as RDI > 1/hour. BMI = Body Mass Index. CBCL (Child Behavior Checklist): T-scores clinical range >69 and subclinical range 65–69. Conner Attention Deficit Hyperactivity Disorder (ADHD) total score: T-Scores>60 are within clinical range for ADHD diagnosis. Pediatric Sleep Questionnaire (PSQ). Mean scores (Standard deviation) are presented for normally distributed data. Otherwise, data is presented as median (min, max).

### Procedures

**Fig 1:**
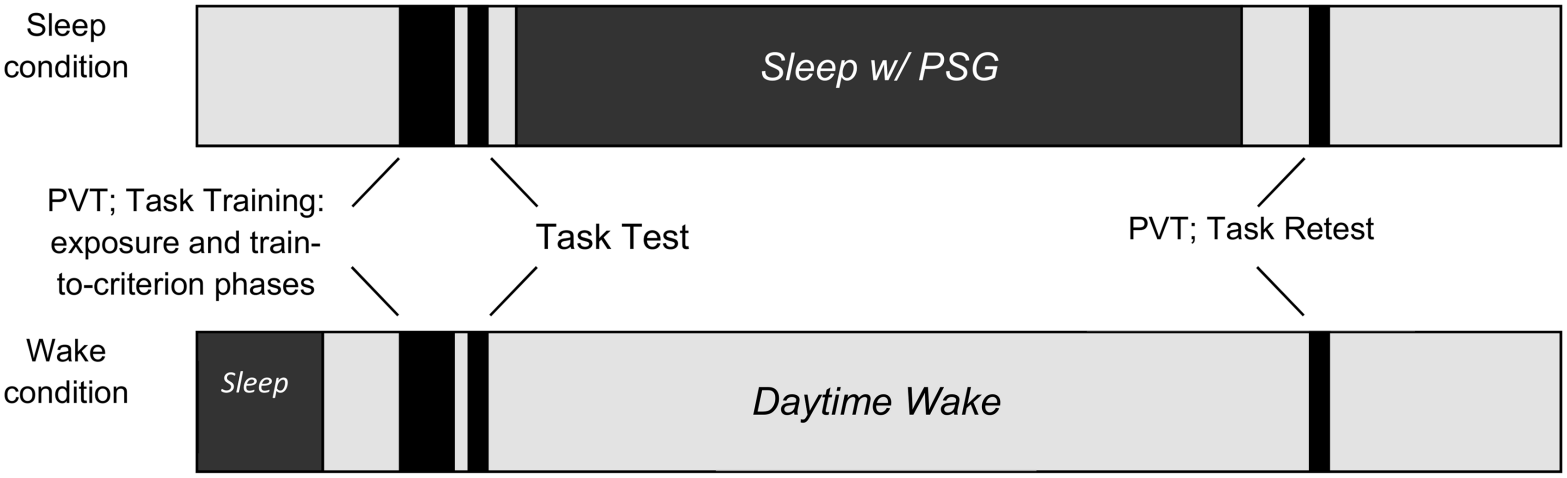
Study procedure. Procedure for memory declarative memory task and psychomotor vigilance test in the Sleep and Wake Conditions. PSG = polysomnogram. PVT = psychomotor vigilance test.

A schematic overview of the protocol is presented in Fig 1. Wake and Sleep conditions were counterbalanced in this study and there were no effects due to order of conditions or task versions presented across conditions. For the Sleep condition, participants came to the Boston Children’s Sleep Lab between 7–8 p.m. where a research assistant administered a psychomotor vigilance test (PVT) and then trained and tested participants on the declarative memory task. Parents/guardians were asked to complete the questionnaires on the night of the sleep study. Sleep technologists then set up participants for an overnight PSG. The PSG was concluded by 6:30 a.m. Approximately 30 minutes after PSG monitoring equipment was removed, the research assistant re-administered the PVT to participants and re-tested them on the declarative memory task. One week before or after the Sleep condition, participants completed the Wake condition. The Wake condition took place in the participants’ homes with a research assistant administering the PVT 30 minutes after the participants’ habitual wake time followed by training and testing on the declarative memory task. Approximately 10 hours later, the research assistant returned to the homes and re-administered the PVT and re-tested the participants on the declarative memory task. Between tests, participants in the Wake condition were asked to proceed with their normal daily activities and parents kept a log of any unusual stresses or changes in routine. Participants were also asked not to nap during the day.

### Measures

#### Questionnaires

Details about the Pediatric Sleep Questionnaire (PSQ) [19], Children’s Behavior Checklist (CBCL) [20] and Conners' Parent Rating Scale–Revised: Short Form (CPRS–R:S) [21] are provided in our prior work [22].

#### Spatial declarative memory task

Sleep-dependent memory processing was measured with a nonverbal, two-dimensional visual spatial memory task that has previously shown sleep-dependent consolidation in children [12,22,23]. The task is similar to the children’s game known as “Memory” or “Concentration,” and consists of 10 colored card pairs with images of animals and common objects arranged in a 5 × 4 array on a laptop computer screen. To avoid floor and ceiling effects, different versions of the task with 6-card pairs and 15-card pairs were also created. Two participants (one from the OSA group and another from PS group) failed to reach criterion after 8 training blocks and were switched to an easier 6-card pair (3x4 array) version of the task. One PS participant who scored 80% on the first trial of the 10-pair task was switched to a more difficult, 15 card-pair (5x6 array) version of the task.

Full details of the task are described elsewhere [22]. Briefly, the task learning occurs in two steps, an exposure phase and a training phase. At the start of the exposure phase, the participants are told to pay attention, and that they will be tested shortly. The screen initially displays the backs of all the cards. Then one card is displayed, followed by its matching pair 1000 ms later. Then, after 3000 ms, both cards are turned face down. This process is repeated until all card pairs have been displayed, and then the entire procedure is repeated a second time. The order of presentation of card pairs is randomized each time the cards are displayed. In the training phase, a block of feedback trials is conducted. The first card of each pair is exposed and participants click on the back of the card they believe matches the exposed card. If they choose the correct card, it is turned over, displaying the matching picture. If they choose an incorrect card, the correct matching card is turned over instead, revealing its location. In either case, the correct pair is left displayed for 2000 ms before being turned back over. After all card pairs have been tested, additional feedback blocks are conducted until participants reach a criterion score of at least 40% of card pairs (*e*.*g*., 4 of 10) correctly identified in one block.

When criterion is reached, the immediate testing phase, consisting of a single block of trials without feedback, is begun. Again, the first card of each pair is exposed, and participants attempt to identify the matching card. In this case, the participant receives no feedback, and the correct card location is not shown. Instead, the back of the selected card is merely highlighted. At the end of the test, the number of correct pairs is displayed. Then, approximately 10 hours later, participants are retested on the task in the identical manner, although again with a randomize ordering of the pairs.

Baseline learning capabilities are reflected by number of trials required to meet criterion on the initial 10-card pair task and by immediate recall during the testing phase (percent of card pairs correctly recalled at the last training trial on the final 6, 10, or 15 card pair task). Memory consolidation is calculated as the percent change in correct card pairs from test to retest sessions [100 x (retest score−immediate test score)/immediate test score].

#### Psychomotor vigilance test (PVT)

A computerized, 3-minute version of the PVT obtained from Psychology Experimental Building Language (http://pebl.sourceforge.net), was used to assess attention prior to memory testing. Participants were instructed to attend to a black computer screen and press a response button whenever a red circle appeared at the center of the screen. The reaction time (RT) in milliseconds was displayed for 1 second after each response. The inter-stimulus interval varied randomly from 2–12 seconds. Participants were instructed to try to keep the RT as low as possible but not press the button too soon (which revealed a message, “You pushed the button too soon!”). Participants were also notified if they did not respond to the signal after a 1second period: “You did not push the button!” Definitions for errors of omission and commission were based on a study of normative PVT performance in children ages 6–11 years [24]. An *omission* was counted whenever the participant failed to press the response button within 500 ms after a stimulus appeared. A *commission* was scored whenever the participant inappropriately pushed the response button before a stimulus appeared (or within 100 ms after it appeared). Performance speed was characterized as the mean reaction time for correct responses across a 3-minute PVT session.

#### Sleep measures from polysomnography (PSG)

PSG was recorded and scored by registered sleep technicians at Boston Children’s Hospital Sleep Labs, and in accordance with American Academy of Sleep Medicine (AASM) Manual for the Scoring of Sleep and Associated Events [25] using an XLTEK^***®***^ PSG system and Natus SleepWorks software (Natus Medical Inc., San Carlos, CA). The PSG studies recorded the following signals: electroencephalogram (EEG), electrooculogram (EOG), submental electromyogram (EMG), electrocardiogram (ECG), thoracoabdominal excursion (plethymography), nasal airflow (pressure transducer and thermistor), snoring sounds (tracheal microphone), pulse oximetry, end tidal carbon dioxide, leg movements and body position. For EEG monitoring, nine EEG channels (F3, F4, T3, T4, C3, Cz, C4, O1, and O2) were recorded digitally at 200 Hz. The PSGs were interpreted by board certified sleep physicians blinded to questionnaire, declarative memory and PVT data.

Sleep latency (SOL, time to fall asleep), wake time after sleep onset (WASO), total sleep time (TST), sleep efficiency (SE, time asleep/time in bed), arousal index, periodic limb movement index, and percentage of total sleep time spent in N1 (NREM 1), N2 (NREM 2), N3 (NREM 3 and 4) and REM sleep stages were collected. An obstructive RDI was calculated based on the number of obstructive apneas, hypopneas and respiratory effort-related arousals per hour of sleep. Obstructive AHI was calculated based on the number of obstructive apneas and hypopneas per hour of sleep.

Sleep recordings were then preprocessed and further analyzed using BrainVision Analyzer 2.0 (Brain Products, Munich, Germany) and MATLAB R2010a (The Math Works, Natick, MA) software. Artifacts including arousals, movement and respiratory events were manually rejected by visual inspection. The EEG was filtered at 0.3 to 35 Hz. Spectral power density was calculated by Fast Fourier Transformation, applying a Hanning window to successive 3 second epochs of NREM sleep (collected from the whole night) with 50% overlap. Spectral power was obtained from and averaged across all 9 EEG channels. Specific spectral power bands calculated for this study were NREM slow wave oscillation (0.5–1 Hz) and N2 sigma (12–15 Hz) frequency ranges. N2 sigma power served as a proxy for N2 sleep spindles [26,27].

### Statistical analyses

Recruitment for this study was targeted to reach power of 85% for P = 0.05 to detect 25% true difference of mean memory consolidation with 15% standard deviation [23] between OSA and control groups. Data were maintained in a REDCap database [28] and analyses performed using SPSS for Windows (version 19; IBM Corp, Armonk, NY, USA). Outcome data were inspected visually to check for normality and potential outliers (points that extend more than 3 times the interquartile range from the edge of box plots). The outcome measure of memory consolidation was normally distributed in both Wake and Sleep conditions across all groups.

The participants’ demographic, questionnaire, and sleep study results are reported as means and standard deviations, and ANOVA tests were applied for group comparisons. For non-normal data, medians with minimum and maximum values are reported, and Kruskal-Wallis tests used for group comparisons. For categorical and ordinal data such as sex and coded maternal education, Fisher exact tests were used for comparisons.

Statistical analysis of memory consolidation was performed using a mixed-effects regression model [within factor is condition (Sleep and Wake); between factor is group; interaction is group x condition] and diagonal covariance matrix, in order to take into account any within-subjects correlations between memory consolidation in Sleep and Wake conditions. Baseline learning capabilities on the task (number of trials to criterion) and immediate recall (post-learning test performance) were assessed with these same mixed-model regression analyses. Similarly, PVT data, including mean reaction times, number of omissions, and number of commissions, were assessed using the mixed model regression analysis. All analyses were adjusted for *a priori* confounders of age and BMI [29]. All learning, memory and PVT data are reported as mean values with standard error margins. Memory task version and order of condition did not significantly affect performance analyses, and hence all analyses were collapsed across these parameters.

Last, to assess any relationships between hypothesized sleep measures (N2 sigma power and NREM slow oscillation power) and the outcome (sleep-dependent memory consolidation), Pearson correlations tests were conducted. Univariate correlations with a P-value < 0.05 were then further examined using linear regression to adjust for confounders of age and BMI. To further determine if these neuro-oscillations played a mediating effect between group and overall memory consolidation, we conducted observed variable path analysis and bootstrap estimation controlling for age and BMI using the PROCESS macro, version 2.16, [30] for SPSS software.

Statistical significance was taken at P < 0.05 level for all output.

## Results

### PSG sleep data

Table 2 shows group sleep findings collected from the nocturnal PSG. The median obstructive RDI was 4.1/hour (1.2, 90) in the OSA group, 0.2 (0,0.7) in PS group, and 0 (0,0.2) among controls (P<0.001). As expected, median group obstructive AHI also differed [OSA: 2 (0.8, 90), PS: 0 (0,0), controls: 0 (0,0.2), P<0.001). No group differences were noted in TST, total arousal index, WASO, sleep efficiency, or sleep stage percentages across the night (P’s>0.16). However, comparison of quantitative EEG data revealed group differences in sigma power in N2 sleep (F = 5.15, P = 0.01). Post-hoc testing showed that participants with OSA have lower N2 sigma power than controls [OSA: 1.19(0.6) vs. controls = 2.17(1.1), P = 0.004] and PS participants [PS: 1.88(0.6), P = 0.03]. N2 sigma power was not significantly different between the PS and controls [P = 0.39]. NREM slow oscillation power was similar between groups (F = 0.37, P = 0.70). All results retained significance even after excluding an outlier in the OSA group with RDI = 90 and controlling for age and BMI.

**Table 2:** Polysomnogram sleep measures.

PSG	OSA n = 14	PS n = 12	Control n = 10	P-value
**Obstructive RDI (events/hour)**	4.1 (1.2,90)	0.2 (0,0.7)	0 (0, 0.2)	**<0.001**
**Obstructive AHI (events/hour)**	2 (0.8, 90)	0 (0,0)	0 (0, 0.2)	**<0.001**
**Percent time snoring in lab (%)**	71.1(30.2)	50.5(27.7)	1.2(3.8)	**<0.001**
**Oxygen nadir (%)**	92.9 (9.6)	98 (3)	99.6(1.2)	**0.03**
**Periodic Limb Movement Index**	2.4 (2.3)	2.8(2.5)	1.9(0.6)	0.67
**SOL (min)**	30.8(16.9)	26.0(13.4)	30.4(21.1)	0.74
**WASO (min)**	25.6(33.5)	24.3(15.4)	17.5(13.1)	0.70
**SE (%)**	88.5(7.8)	87.6(7.4)	90.4(4.8)	0.65
**TST (min)**	436.2(42.5)	424.3(48.3)	455.7(32.4)	0.23
**REM (%)**	20.7(5.3)	19.7(6.2)	21.2(4)	0.80
**N1 (%)**	5.8(3.1)	5.6(3.7)	4.9(3.4)	0.79
**N2 (%)**	37.5(7.3)	41.4(9.6)	34.8(6.6)	0.16
**SWS (%)**	36.0(6)	33.2(9.9)	39.2(7.9)	0.23
**Arousal Index (events/hour)**	14.1(12.4)	17.6(31.7)	9.4(3.4)	0.63
**N2 Sigma Power (μV**^**2**^**Hz**^**-1**^**)**	1.19(0.6)	1.88(0.6)	2.17(1.1)	**0.01**
**NREM Slow Oscillation Power (μV**^**2**^**Hz**^**-1**^**)**	1.21(0.6)	1.20(0.4)	1.06(0.3)	0.70

Sleep Architecture data obtained from PSG night. RDI = Respiratory Disturbance Index. Obstructive AHI = Obstructive Apnea Hypopnea Index. Oxygen nadir is the lowest oxygen value recorded during the night. SOL (sleep onset latency), WASO (wake after sleep onset), SE (sleep efficiency), TST (total sleep time), N1 (stage 1 NREM sleep), N2 (stage 2 NREM sleep), SWS (N3+N4 NREM sleep), N2 Sigma power (12–15 Hz) in stage 2 NREM sleep, Slow Oscillation power (0.5–1 Hz) over NREM sleep. Mean (stand deviation)

### Task learning and memory consolidation

**Fig 2:**
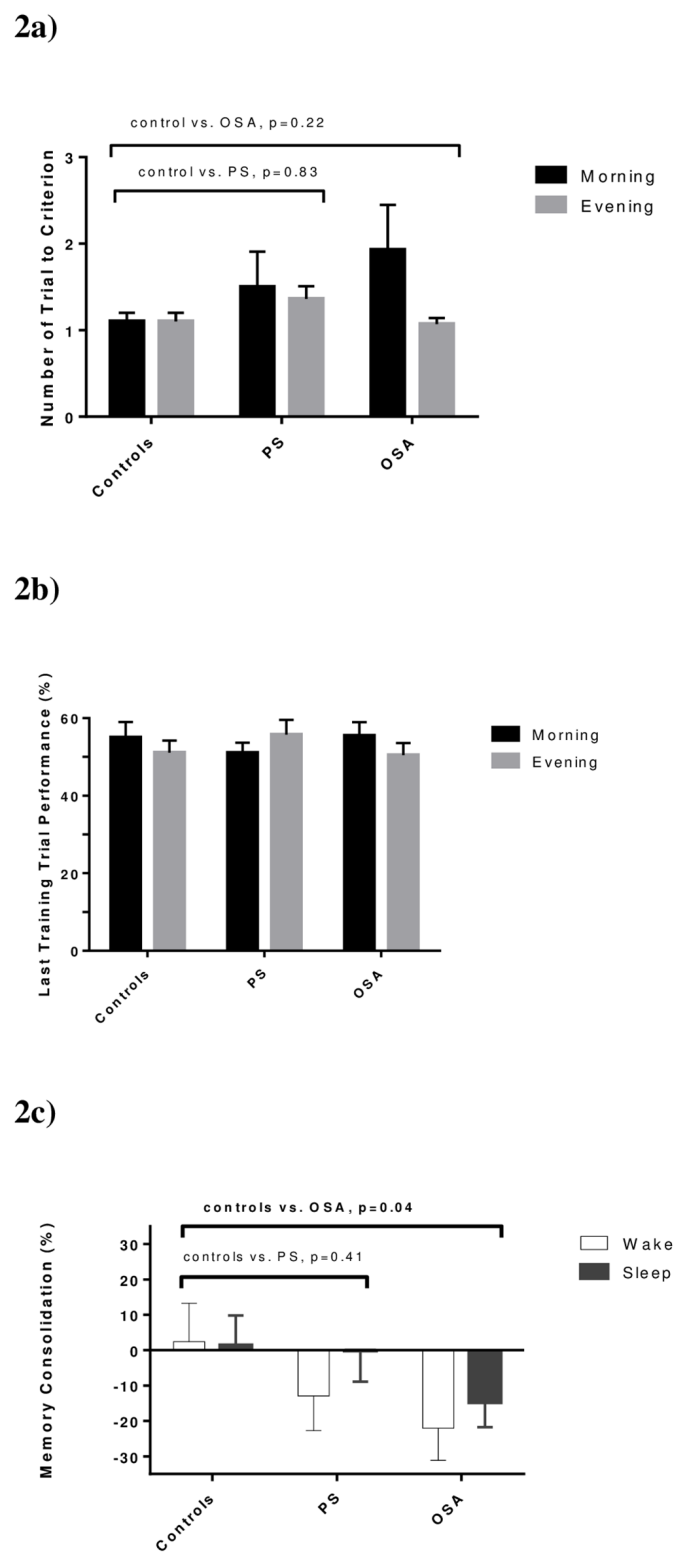
Declarative memory task results in wake and sleep conditions. 2a) Training: Learning performance reflects number of criterion blocks (number of training block to reach criterion for testing on initial 10 card pair task) in morning and evening condition. PS and OSA groups had comparable results to controls in post-hoc tests. 2b) Initial Recall: Immediate recall is accuracy on last criterion block of the training phase and is also evaluated in morning and evening conditions. Here too no main effects for group or condition were noted. 2c) Delayed Recall: Delayed recall reflects memory consolidation processes. This dependent variable reflects relative change in recall accuracy (retest-immediate test/immediate test score) evaluated in wake and sleep conditions. On mixed regression analysis, only group differences in memory consolidation were noted between controls and OSA participants adjusting for age and BMI. Mean with standard error margins are displayed.

On the initial 10 card-pair task, groups took a similar number of trials to reach criterion [mean number of trials: OSA = 1.5(0.26), PS = 1.43(0.22), controls = 1.1(0.07), P’s>0.20, (Fig 2a)]. Adjusting from age and BMI, there was no main effect for group (F = 1.63, P = 0.21) but participants had an easier time learning (fewer trials to criterion) in the evening [1.18(0.7) trials] compared to the morning [1.52(0.24); F = 5.41, P = 0.03]. Fig 2a demonstrates that this condition effect was most notable for OSA participants and to a lesser extent for the PS group but controls required similar number of trials to reach criterion in the morning or evening. No interaction between group and time of training (morning, evening) was detected (F = 1.16, P = 0.33).

Fig 2b shows last training trial performance, which also reflects participants’ learning capabilities. Across conditions, participants recalled an average 53.2% of card pairs. We detected no main effects for group (F = 0.23, P = 0.80), main effect for time of testing (morning vs. evening: F = 0.65, P = 0.43), or interaction between group x time of testing (F = 1.76, P = 0.19).

Fig 2c shows the memory consolidation across Wake and Sleep for each group. Across conditions, participants showed an average 8.16% decrease in recall from initial test to retest approximately 10 hours later. Adjusting for age and BMI, group differences in mean memory consolidation neared significance (mean memory consolidation: OSA = -18.7% (5.8); PS = -6.5% (6.3), controls = 1.9% (7.2); F = 2.49, P = 0.09]. Given that our main aim was to compare memory consolidation between OSA and control groups, we proceeded with post-hoc comparisons. Participants with OSA showed significantly more forgetting than controls [*B* = -20.2(9.5), 95 CI (-37.3 to -1.78), P = 0.04]. With PS participants’ scores lying between those of OSA and control groups, no difference in memory consolidation was detected between PS and controls [*B* = -8.4(10.1), P = 0.41] or between PS and OSA participants [*B* = -11.9(8.5), P = 0.17]. Looking at performance across Wake and Sleep separately, neither condition showed significant differences across groups (P’s >0.21). Of note, participants’ memory consolidation did not differ across Wake and Sleep conditions as predicted [memory consolidation: Wake = -10.6(5.7), Sleep: -4.6(4.5); F = 0.78, P = 0.38]; no group x condition interaction was found (F = 0.28, P = 0.75).

### Sleep measures and memory consolidation

**Fig 3:**
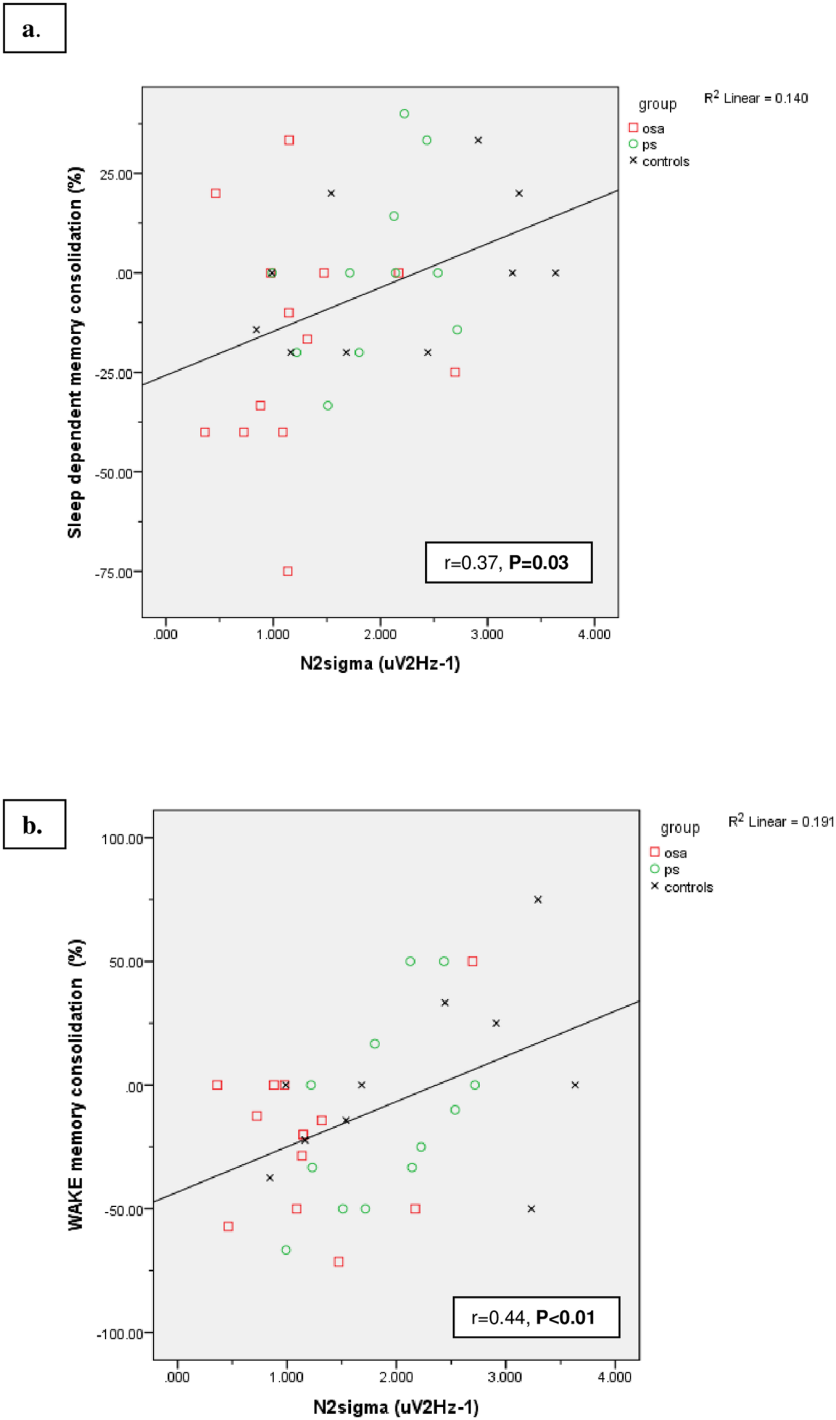
Correlations between N2 sigma power and memory consolidation. Significant positive correlations were detected between N2 sigma power and memory consolidation in both sleep **(a)** and wake **(b)** conditions across all participants. Results retained significance adjusting for BMI and age.

Pearson correlations were used to assess relationships between specified sleep PSG parameters of interest (N2 sigma power, NREM slow oscillation power) and sleep-dependent memory consolidation. On univariate testing with all participants, sleep-dependent memory consolidation positively correlated with N2 sigma power (r = 0.37, P = 0.03) and results retained significance adjusting for BMI and age [F = 2.18, P = 0.03, partial Eta squared = 0.15; (Fig 3a)]. Importantly, we found no association between participants’ N2 sigma values and trials to criterion (r = 0.09, P = 0.63) nor last learning trial performance in the evening (r = 0.03, P = 0.85) which would have suggested that N2 sigma reflected baseline learning capabilities. However, we did not find any within-group correlations between sleep-dependent memory consolidation and N2 sigma power (OSA: r = 0.08, P = 0.81; PS: r = 0.41, P = 0.21; controls: r = 0.43, P = 0.22). We did not detect associations between sleep-dependent memory consolidation and NREM slow oscillation power across participants or within groups (P’s>0.7).

On further exploratory analyses, correlations between N2 sigma and percentage of sleep time technicians reported participant snoring on the study night reached significance (r = -0.33, P = 0.047). We did not detect associations between the predictors arousal index, oxygen nadir, WASO, AHI, or RDI with outcome measures of N2 sigma power, wake memory consolidation or sleep memory consolidation (all P’s >0.09).

Given our findings that participants with OSA had reduced memory consolidation across both Wake and Sleep conditions compared to controls and had reduced N2 sigma power compared to both control and PS groups, we further explored whether group differences existed between N2 sigma and memory consolidation across the Wake condition. Similar to the sleep condition, N2 sigma correlated with memory consolidation measured across the waking period [r = 0.44, P = 0.009, (Fig 3b)] but not with trials to criterion (r = -0.08, P = 0.67) nor last training trial performance (r = -0.02, P = 0.32) measured in the morning. Again, we found no within group correlations between N2 sigma and memory consolidation in the Wake condition (P’s> 0.22). All results retained significance adjusting for age and BMI. Taken together, our results suggest that N2 sigma power is positively associated with memory consolidation but not task learning in children.

**Fig 4:**
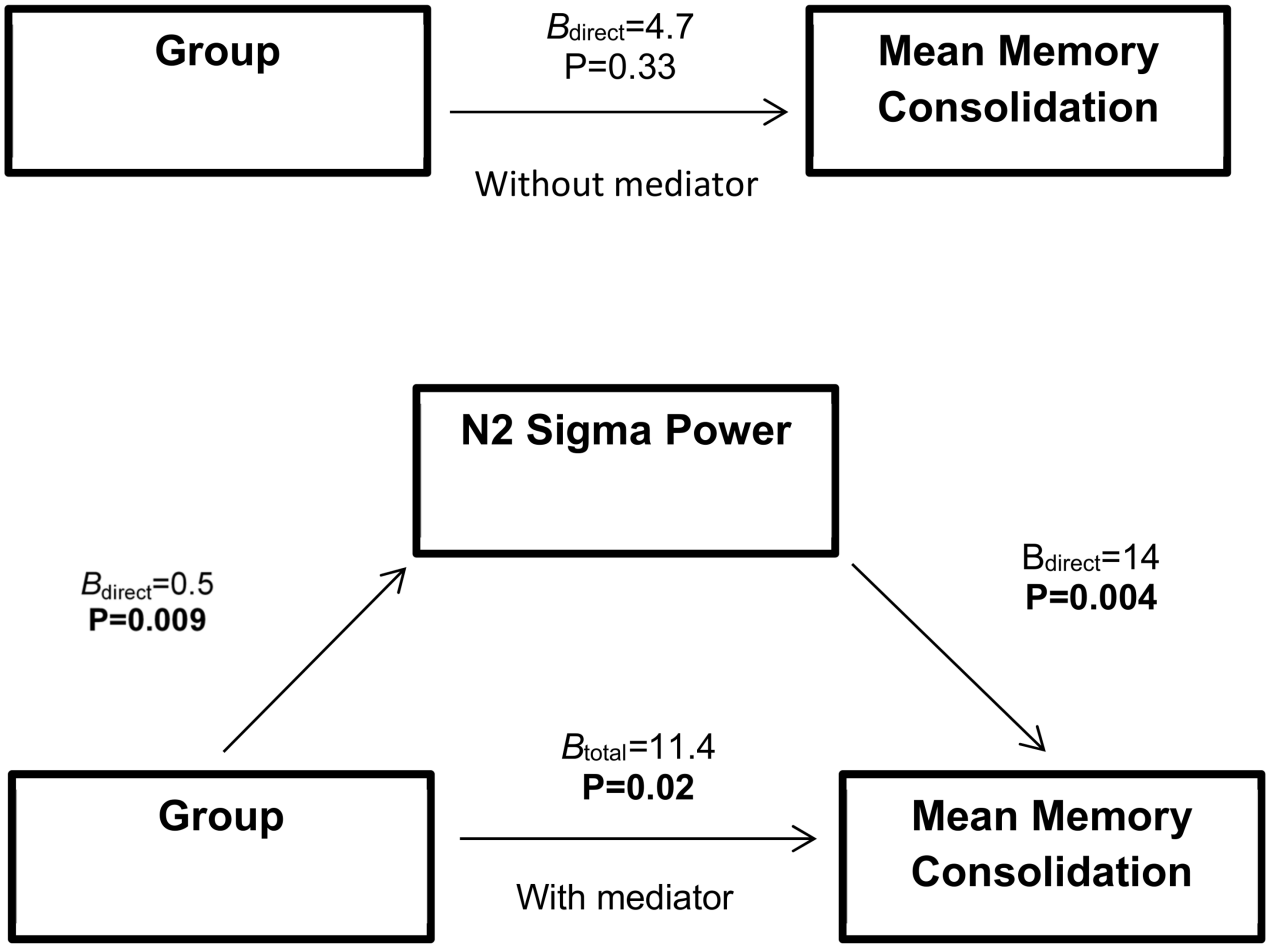
N2 sigma power mediates the relationship between group and memory consolidation. N2 sigma power mediates the relationship between mean memory consolidation across Wake and Sleep conditions. Model was adjusted for BMI and age.

To determine if NREM N2 sigma plays a mediating role between sleep disordered breathing and mean memory consolidation across Wake and Sleep conditions, we conducted observed variable path analysis controlling for age and BMI (Fig 4). In this model, group is associated with mean memory consolidation through N2 sigma [group: *B*_total_ = 11.43(4.8), 95 CI (1.6 to 21.3), t = 2.38, P = 0.02] but the direct effect of group on mean memory consolidation was not significant [group: *B*_direct_ = 4.67 (4.7), t = 0.99, P = 0.33]. Group designation predicts N2 sigma power [group: *B*_direct_ = 0.48 (.17), 95 CI (0.13 to 0.83), t = 2.9, P = 0.009] and N2 sigma power robustly predicts mean memory consolidation [N2 sigma power: *B*_direct_ = 14.04 (4.5), 95 CI (4.9 to 23.2), t = 3.1, P = 0.004]. Notably, this analysis shows that N2 sigma power mediates the relationship between group and mean memory consolidation (*B*_indirect_ = 6.76(3.5), 95 CI (1.7 to 16.1), z = 2.03, P = 0.04).

### PVT data

There were no main effects for group or time-of-day and no group x time interactions for mean reaction time, omissions or commissions (P’s >0.11). These findings suggest that attention was not different between groups nor were there circadian effects on attention at time of task learning or retesting.

## Discussion

Our study demonstrates impaired memory consolidation in children with mild OSA (defined as RDI >1/hour) compared to controls, a global deficit seen across Wake and Sleep conditions. Notably, we found that OSA participants had less N2 sigma power compared to other groups, N2 sigma power correlates with amount of snoring on the study night, and memory consolidation is predicted by N2 sigma power. We further show that N2 sigma power has a mediating effect between SDB and mean memory consolidation across Wake and Sleep conditions. We found no correlations between N2 sigma and number of trials to criterion or last training trial performances in Wake or Sleep conditions to suggest that N2 sigma reflects baseline learning capabilities.

In this study, we use N2 sigma power as a proxy for sleep spindles and both of these electrophysiologic measures have demonstrated associations with sleep-dependent memory consolidation in adults and children [12,27,31]. It has been unclear in the literature whether N2 sigma power and sleep spindles represent individual traits associated with learning and memory functions or whether they actually play a causal role in memory consolidation. Supporting trait-like features of sleep spindles, Hoedlmoser et al. reported that NREM stage 2 and 3 sleep spindle activity (11–13 Hz) was associated with general baseline cognitive abilities and learning capabilities on a declarative memory task but not sleep-dependent declarative memory consolidation [32]. Our study cannot exclude the possibility N2 sigma power reflects baseline cognition because we did not measure intellectual quotients. However, we did not find associations with our learning measures (numbers of trials to meet criterion and last training trial score).Notably, our results show that N2 sigma correlates with memory consolidation in both Wake and Sleep conditions across all participants suggesting that N2 sigma could be a trait biomarker of memory consolidation capabilities in children.

On the other hand, given that N2 sigma power varies with SDB diagnoses and snoring and mediates the relationship between group and mean memory consolidation across Wake and Sleep, our data support that this neuro-oscillation has state-like characteristics that facilitate offline memory processing when undisturbed (as in controls). In adult patients, arousals (but not AHI or oxygen desaturations) from sleep have been shown to predict the relationship between OSA and sleep-dependent memory consolidation [13] but we found no such association in our pediatric cohort. This may be because children have a higher arousal threshold compared to adults and thus their sleep architecture is more protective against sleep fragmentation [33]. Lack of association between AHI, RDI, oxygen nadir and arousal index and memory consolidation in this study may explain why there are inconsistent findings showing relationships between pediatric sleep disordered breathing disease severity and other cognitive outcomes in the literature [7]. Ultimately, N2 sigma power may be a better predictor of the cognitive effects of SDB in children than standard respiratory measures.

Our findings contrast with data from studies using standard neuropsychological testing that show no associations between any level of sleep disordered breathing (primary snoring, mild-moderate OSA, and severe OSA) and IQ [34,35]. However, the sensitivity of neuropsychological batteries has been questioned as research has more consistently shown that children with SDB have poorer academic grades and learning problems as reported by parents and teachers [5,36]. Memory consolidation testing may produce more variation in results than IQ testing and be a more sensitive outcome measure to assess cognitive effects of SDB. Additionally, we excluded children with diagnosed developmental and learning disorders and our study design allowed us to control for factors that may influence memory consolidation testing such as sustained attention, learning capabilities, and circadian factors. Here, group differences only emerge on testing of memory consolidation. Neuroimaging studies have shown that children with moderate to severe OSA have abnormal cortical grey matter volume [37] and neuronal metabolites in the hippocampi [38]. Future neuroimaging studies are needed to determine neural correlates of milder SDB so as to clarify mechanisms that underlie impaired memory consolidation in children.

Counter to our expectations, we did not find evidence that Sleep resulted in better memory consolidation than then testing in the Wake condition as shown in prior our research using a similar declarative memory task [22]. Participants in our current study slept in a diagnostic sleep laboratory, whereas our prior study was conducted with home-based PSG recordings and with no respiratory monitoring. In the laboratory setting, the environment is unfamiliar to the child, more equipment is involved in monitoring, and technician interventions are necessary to replace leads and reposition the child to ensure good evaluation of sleep disordered breathing. It is possible that the inevitable decrease in sleep quality in this environment diminished the enhancement of memory across Sleep in all participants. Furthermore, we did not find a predicted effect between NREM slow oscillations and memory consolidation. This may be because we did not analyze slow oscillation power specifically in slow wave sleep. In adults, sleep-dependent declarative memory consolidation is causally linked to slow oscillation activity (<1 Hz) during slow wave sleep [39,40]. We chose to test slow wave oscillation power in NREM sleep because prior research has not demonstrated a relationship between memory consolidation and slow wave sleep in children [12,23,41]. Healthy children show a correlation between NREM slow oscillation power and emotional memory consolidation in an older cohort with mean age of 12–13 years[15]. Thus, NREM slow oscillation may be more predictive of emotional memory processes among children in general or more important for declarative memory processes as children age into adolescence.

There are limitations in this study. For one, our recruitment numbers were small. Most OSA participants had mild OSA (median AHI = 2/hour) and thus their sleep architecture and memory processes may not be that distinct from PS. Consequently, we may be underpowered to detect group differences in sleep measures, group and condition interactions, and the direct effect of group on memory consolidation. Nevertheless, we found significant differences in our primary outcome of memory consolidation between children with OSA and controls. Secondly, selection bias is possible as participants with OSA and PS came from a clinical referred population for an overnight PSG and most controls were recruited from the community. As such, there may have been some concern about undiagnosed learning and memory in clinically referred children that prompted a clinician to order the sleep study. Thirdly, as mentioned above, neurocognitive testing was not performed on participants to ensure similar cognitive capacities. Overall, this study requires validation with larger cohorts with a wider spectrum of SDB who are matched for baseline IQ.

## Conclusion

In conclusion, this study provides needed evidence that memory processes are disrupted at even low severity levels of SDB and suggests that treatment of mild OSA could be beneficial for memory processes in children. Additionally, assessment of memory consolidation may provide a dynamic evaluation of memory processes that can compliment neuropsychological and neuroimaging studies in future research on SDB and cognition. Finally, this study demonstrates that N2 sigma power mediates the relationship between SDB and memory consolidation. Given its state-like characteristics, N2 sigma power could be a clinically useful neurophysiological biomarker of functionally relevant disrupted sleep in children with SDB.
